# Estimated cost of asthma in outpatient treatment: a real-world study

**DOI:** 10.11606/S1518-8787.2018052000153

**Published:** 2018-03-14

**Authors:** Eduardo Costa, Rosangela Caetano, Guilherme Loureiro Werneck, Maurício Bregman, Denizar Vianna Araújo, Rogério Rufino

**Affiliations:** I Universidade do Estado do Rio de Janeiro. Faculdade de Ciências Médicas. Departamento de Medicina Interna. Rio de Janeiro, RJ, Brasil; II Universidade do Estado do Rio de Janeiro. Instituto de Medicina Social. Departamento de Política, Planejamento e Administração em Saúde. Rio de Janeiro, RJ, Brasil; III Universidade do Estado do Rio de Janeiro. Instituto de Medicina Social. Departamento de Epidemiologia. Rio de Janeiro, RJ, Brasil; IV Universidade do Estado do Rio de Janeiro. Faculdade de Ciências Médicas. Programa de Bolsas de Iniciação Científica. Rio de Janeiro, RJ, Brasil; V Universidade do Estado do Rio de Janeiro. Faculdade de Ciências Médicas. Departamento de Clínica Médica. Rio de Janeiro, RJ, Brasil; VI Universidade do Estado do Rio de Janeiro. Faculdade de Ciências Médicas. Departamento de Doenças do Tórax. Rio de Janeiro, RJ, Brasil

**Keywords:** Asthma, economics, Anti-Asthmatic Agents, supply & distribution, Sick Leave, Health Care Costs, Costs and Cost Analysis, Ambulatory Care, Clinical Decision-Making, Asma, economia, Antiasmáticos, provisão & distribuição, Licença Médica, Custos de Cuidados de Saúde, Custos e Análise de Custo, Assistência Ambulatorial, Tomada de Decisão Clínica

## Abstract

**OBJECTIVE:**

To estimate the cost of diagnosis and treatment of asthma.

**METHODS:**

We used the perspective of society. We sequentially included for 12 months, in 2011-2012, 117 individuals over five years of age who were treated for asthma in the Pneumology and Allergy-Immunology Services of the Piquet Carneiro Polyclinic, Universidade do Estado do Rio de Janeiro. All of them were interviewed twice with a six-month interval for data collection, covering 12 months. The cost units were identified and valued according to defined methods. We carried out a sensitivity analysis and applied statistical methods with a significance level of 5% for cost comparisons between subgroups.

**RESULTS:**

The study consisted of 108 patients, and 73.8% of them were women. Median age was 49.5 years. Rhinitis was present in 83.3% of the individuals, and more than half were overweight or obese. Mean family income was U$915.90/month (SD = 879.12). Most workers and students had absenteeism related to asthma. Total annual mean cost was U$1,291.20/patient (SD = 1,298.57). The cost related to isolated asthma was U$1,155.43/patient-year (SD = 1,305.58). Obese, severe, and uncontrolled asthmatic patients had higher costs than non-obese, non-severe, and controlled asthmatics, respectively. Severity and control level were independently associated with higher cost (p = 0.001 and 0.000, respectively). The direct cost accounted for 82.3% of the estimated total cost. The cost of medications for asthma accounted for 62.2% of the direct costs of asthma.

**CONCLUSIONS:**

Asthma medications, environmental control measures, and long-term health leaves had the greatest potential impact on total cost variation. The results are an estimate of the cost of treating asthma at a secondary level in the Brazilian Unified Health System, assuming that the treatment used represents the ideal approach to the disease.

## RESUMO

**OBJETIVO:**

Estimar o custo do diagnóstico e tratamento da asma.

**MÉTODOS:**

Foi utilizada a perspectiva da sociedade. Foram incluídos por 12 meses em 2011–2012, sequencialmente, 117 indivíduos maiores de cinco anos de idade, em tratamento por asma nos Serviços de Pneumologia e Alergia-Imunologia da Policlínica Piquet Carneiro, Universidade do Estado do Rio de Janeiro. Todos realizaram duas entrevistas com seis meses de intervalo para coleta de dados, cobrindo 12 meses. As unidades de custos foram identificadas e valoradas de acordo com métodos definidos. Foi feita análise de sensibilidade e foram aplicados métodos estatísticos com nível de significância de 5% para comparações de custos entre subgrupos.

**RESULTADOS:**

Cento e oito pacientes completaram o estudo, 73,8% eram mulheres. Mediana de idade foi de 49,5 anos. Rinite esteve presente em 83,3%, e mais da metade tinha sobrepeso ou obesidade. A renda familiar média foi de R$1.566,19/mês (DP = 1.503,30). A maioria dos trabalhadores e dos estudantes teve absenteísmo relacionado à asma. O custo médio anual total foi de R$2.207,99/paciente (DP = 2.220,55). O custo relacionado à asma isolada foi de R$1.984,17/paciente-ano (DP = 2.232,55). Asmáticos obesos, graves ou não controlados tiveram maiores custos em comparação aos não obesos (p = 0,001), não graves e controlados (p = 0,000). O custo direto correspondeu a 82,3% do custo total estimado. O custo com medicamentos para asma correspondeu a 62,2% dos custos diretos da asma.

**CONCLUSÕES:**

Medicamentos para asma, medidas de controle ambiental e licenças de saúde prolongadas tiveram maior impacto potencial na variação do custo total. Os resultados são uma estimativa do custo do tratamento da asma em nível secundário no Sistema Único de Saúde, assumindo-se que o tratamento utilizado represente a abordagem ideal da doença.

## INTRODUCTION

Asthma is a heterogeneous disease defined by the occurrence of variable and intermittent symptoms. It is characterized by the chronic inflammation of the airway associated with variable airflow limitation. The prevalence of asthma has increased in the developed world in the last decades of the 20th century and it is frequently associated with chronic rhinitis^6^ ^,^ ^a^.

The International Study of Allergies and Asthma in Childhood (ISAAC) has found an average prevalence of 12.6% and 14.6% of allergic rhinitis in children and adolescents in Brazil, respectively, and prevalence of asthma of 24.3% and 19.0% in the same groups^24^. A study showed a prevalence of 13.1% of asthma in Brazilian adolescents in 2013-2014^15^ and data from the southern region of the country pointed to the prevalence of 5.2% of asthma in adults in 2010^8^. A Latin American study based on a telephone survey has estimated the prevalence of asthma in Brazil in 13.3%^12^. The latest national guidelines on asthma suggest the existence of 20 million asthmatic persons in the country^23^.

Asthma has a significant cost to health systems around the world because of its high prevalence. A study has analyzed data from 47,033 American adults treated in the health system between 2003 and 2005 and it has estimated a total medical expenditure in the country of US$18 billion^26^. Van Den Akker-van Marle et al. have estimated a total annual direct cost for childhood asthma and wheezing of €3 million in the 25 participating European countries, reaching €5.2 million when projected for the entire European Union (EU)^28^. For adults, the average annual cost was €1,583/patient, with an estimated total cost of €4.3 billion for the eleven studied countries and €19.3 billion for the entire region^2^. The total cost of the disease in the Asia-Pacific region was equivalent to 13.0% of the gross domestic product (GDP) of the region, while in the USA it was 2.0%^26^. However, the different methodologies used in the studies do not allow us to compare directly these proportions among countries.

Data on the costs of asthma in Brazil are limited, with few published studies mainly addressing severe cases, because of their economic impact from the greater use of hospital resources^13^ ^,^ ^22^. However, the World Health Organization (WHO) estimates that only 5.0% to 10.0% of asthmatics have the severe form of the disease^a^. Fortunately, the number of asthma hospitalizations in the Brazilian Unified Health System (SUS) has decreased in recent years, and this positive result probably reflects the greater use of inhaled corticosteroids, given its free dispensation in the SUS^b^. However, the cost data of asthma in outpatient treatment addressing the different levels of disease severity and control are scarce in Brazil.

The objective of this study was to estimate the costs associated with the diagnosis and treatment of asthma in the real world scenario. These data may aid the decision of health policy makers in the allocation of resources for the research and care of asthmatics in the SUS, considering an approach considered ideal for the disease.

## METHODS

This is a study of the cost of disease, based on the prevalence of asthma and on the perspective of society. We collected data regarding direct and indirect costs. The cost units and their amounts were estimated from the primary data collection.

We included patients aged six years and older, with clinical and functional diagnosis of persistent asthma (according to established criteria)^23^ ^,^ ^a^ in follow-up at specialized services in asthma (allergy-immunology and pneumology) at a university outpatient unit in the city of Rio de Janeiro, State of Rio de Janeiro, Brazil, from April to October 2011. We excluded patients who dropped out of treatment (no return for more than four months) and those with a chronic disease that could cause asthma-like respiratory symptoms.

Patients underwent routine clinical visits with three to four months of interval and two interviews for data collection with an interval of six months, performed by two professionals who did not participate in the care. The data collected comprised 12 months (April/2011 to March/2012), covering all climatic seasons. We used a structured and standardized instrument, previously submitted to a pre-test. The interview was answered by those responsible for patients under 18 years of age.

The primary variables were related to the cost of routine visits (with physician, nurse, or physiotherapist) and emergency visits, days of hospitalization, additional examinations, medications used for asthma, rhinitis, and respiratory infections (RI), use of immunotherapy, transportation costs, aeroallergen control measures (environmental hygiene), lost hours of work for treatment, and number of days absent from work or school. In order to avoid memory bias, we doubled the data for the three months before the collection to estimate the expenses of the semester. We excluded the number of days or place of hospitalization and expenses with environmental hygiene, which were easily remembered for the entire period. We obtained the indirect cost estimate from the human capital approach, as the studied population had great occupational heterogeneity and this method is adequate. The cost units, sources, and valuation method, using the bottom-up approach, are defined in Box. All of them were considered as exclusive costs of asthma, excluding costs with medications for rhinitis and respiratory infections (RI). The secondary variables were age, sex, education level, monthly family income (MFI), body mass index (BMI), asthma severity, and asthma control. Asthma severity and control were classified according to GINA^a^. Overweight and obesity were defined by the criteria of the WHO^9^.

**Box:** : Cost units of asthma in outpatient treatment and valuation methods.

Appointments (medical, nursing, or physiotherapy), emergency care, hospitalizations (room, ward, or intensive care), and additional examinations	
Brazilian Unified Health System (SUS)	Outpatient and hospital information system of the SUS – SIA and SIH/SIGTAP/DATASUS^a^
Supplementary Health System (SSS)	Brazilian hierarchical classification of medical procedures (CBHPM) – version 2012^b^
Private system	Value informed by the patient/guardian

Medications for asthma, rhinitis, and respiratory infections	

Provided by the SUS	Table of prices of medications for public purchases of the Executive Department of the National Health Surveillance Agency (ANVISA), updated in March/2012^c^
Funded by families	Table of prices of medications for sale with maximum price to the consumer of the Executive Department of the National Health Surveillance Agency (ANVISA), updated in March/2012^c^. We used the mean price of three different formulations for each drug used
Immunotherapy (vaccines for allergy)	
Bottle	Acquisition value informed by the Financial Division of the Unit – April 2012
Administration (all units of the SUS)	Outpatient and hospital information system of the SUS – SIA and SIH/SIGTAP/DATASUS^a^

Environmental control measures	

Items purchased (covers, curtains, air filters) and expenses for works on flooring, painting, and change of furniture	Value informed by the patient/guardian

Transportation to the health unit for care and exams	

Public transportation or taxi	Value informed by the patient/guardian
Private vehicle or social assistance vehicles of the SUS	The cost was estimated using the value of 1 liter of gasoline for each 10 km of distance traveled The distances between the starting point and the destination were obtained from the website Google Maps^d^ We used the mean gas price of March/2012 provided by the National Agency of Petroleum (ANP)^e^

Absenteeism	

School	Number of days (not valued)
Work (leaves up to 15 days)	Value of each day = informed monthly income of the individual/21 days Cost = value of each day x number of days lost
Work (Sickness benefit [ADB] leaves > 15 days)	Value of each month = informed monthly income of the individual Cost = monthly income x number of months of leaves

SUS: Brazilian unified health system; SAI: outpatient information system; SIH: hospital information system; SIGTAP: management system of the table of procedures, medicines, and OPM (orthotics, prosthetics, and materials) of the SUS; DATASUS: information technology department of the SUS; SSS: supplementary health system; CBHPM: Brazilian hierarchical classification of medical procedures

^a^ Table of procedures - outpatient and hospitalization SIA and SIH – SUS; 2014 [cited 2017 Dec 1]. Available from: http://sigtap.datasus.gov.br/tabela-unificada/app/sec/inicio.jsp

^b^ Brazilian hierarchical classification of medical procedures (CBHPM); 2012 [cited 2014 Oct 21]. Available from: http://www.acm.org.br/cbhpm-2012/

^c^ National Health Surveillance Agency (ANVISA - BR). Brasília (DF), Brazil. Table of prices of medications for public purchases of the Executive Department of ANVISA; 2012 [cited 2014 Oct 21]. Available from: http://portal.anvisa.gov.br/cmed

^d^ Google. Google Maps. Distances in KM between the starting point (residence of the patient) and the destination (health unit); 2014 [cited 2017 Dec 1]. Available from: https://www.google.com.br/maps/place/Av.+Mal.+Rondon,+381+-+S%C3%A3o+Francisco+Xavier,+Rio+de+Janeiro+-+RJ,+20950-003/@-22.9061884,-43.2795421,13z/data=!4m5!3m4!1s0x997e7d1d6deb6f:0x43bf435b497372f9!8m2!3d-22.9061884!4d-43.2445232

^e^ National Agency of Petroleum, Natural Gas, and Biofuels (ANP - BR) – Rio de Janeiro (RJ), Brazil; 2012 [cited 2017 Dec 1]. Historical series of the surveys of fuel prices and sales margins. Available from: http://anp.gov.br/wwwanp/precos-e-defesa-da-concorrencia/precos/levantamento-de-precos/serie-historica-do-levantamento-de-precos-e-de-margens-de-comercializacao-de-combustiveis

All economic variables were quantified in Brazilian Reais (R$). For comparison with international data, we converted the values to US dollars by purchasing power parity (PPP) in 2012 (US$1.00 = R$1.71)^c^. The mean annual cost of asthma was stratified by sex, age, MFI, asthma severity and control, nutritional status (normal weight, overweight, or obesity), and presence of rhinitis. We used the chi-square test (with Fisher’s correction, when necessary) and the Mann-Whitney test to compare the differences between categorical variables and between continuous variables, respectively. Differences between subgroups by levels of severity, control, and nutritional status were analyzed by bivariate and multiple regression methods. In the sensitivity analysis, given the large dispersion of data, we used the 25th-75th percentile values (P_25-75_) in relation to the median of the cost units (appointments, hospitalization, additional examinations, medications for asthma, rhinitis, and RI, immunotherapy, transportation, environmental hygiene, absenteeism, and sickness benefit [BAD]) to calculate the impact of the variation of each of them on the total cost.

Data was entered into Microsoft® Office Excel 2010 spreadsheets (Microsoft Corp., CA, USA) for the descriptive analysis of general characteristics and costs. For the bivariate analysis, we used the program GraphPad Prism 6.0 (GraphPad Software Inc., CA, USA). For the multiple analysis, we performed a logistic regression with logarithmic transformation using the package Stata 11 (Stata Corp, TX, USA). We adopted a confidence level of 5%.

Patients (or their guardians) signed an informed consent. The study was approved by the local Research Ethics Committee and registered with the National Research Ethics Committee (CEP/HUPE 2904/2011 – CAAE 0054.0.228.000-11).

## RESULTS

We included 117 patients and 7.7% did not complete the follow-up. The studied population (n = 108) represented 33.1% of the asthmatic patients being treated at the unit during the year; 73.2% were female, predominantly adults, and median age of 49.5 years (P_25-75_ = 27.7–60.0). Most of them (70.4%) had elementary education, 23.1% had high school, and 6.5% had higher education. The MFI was U$915.90 (SD = 879.12). The MFI was lower among patients with uncontrolled asthma compared to those with partially or fully controlled asthma in both semesters (p = 0.005 and p = 0.01, respectively). The median duration of asthma was 18 years (P_25-75_ = 9.5–33.0). At the time of inclusion in the study, 49.1% of the patients had asthma classified as mild, 36.1% as moderate, and 14.8% as severe. In the final evaluation, the classification of 38.9% of the patients changed to intermittent asthma, and 38.1% of the 21 patients with uncontrolled asthma had better control. Seventy-four patients (68.5%) were overweight or obese. Rhinitis was present in 83.0% of the patients and 28.0% of them used antibiotics for treatment of RI during the study.

The standard deviation greatly varied in relation to the mean values of some cost units, pointing to the high dispersion in these items (Table 1). The cost of asthma was significantly higher in patients without rhinitis (p = 0.03), in adults (p = 0.000), in the more severe cases (p = 0.000), in patients with worse asthma control (p = 0.000), and in those overweight or obese (p = 0.001). The cost of medication for asthma was also higher in patients with more than 20 years of disease (p = 0.008) (Table 2). Direct costs accounted for 82.3% of the total cost, and 49.7% of the exclusive costs of asthma corresponded to medication costs, which is equivalent to 62.2% of the exclusive direct costs of asthma (Table 1).

**Table 1:** Results of the valuation of the cost units of asthma in outpatient treatment – total, mean, and median in the first and second study periods.

Cost units	1st period		2nd period		Total	Mean^a^	SD	Median^b^	P25	P75
							
	n	U$	n	U$	U$	U$	U$	U$	U$	U$
Direct costs										
Scheduled medical visits	444	3,017.54	331	1,986.54	5,004.08	46.33	53.82	35.09	35.09	46.78
Unscheduled medical visits/Emergency^c^	213	2,209.63	99	800.03	3,009.67	27.86	86.92	29.17	14.58	58.34
Nursing care/Physiotherapy^d^	216	630.52	202	586.12	1,216.65	11.26	27.51	25.79	22.11	31.32
Visits (total)	873	5,876.42	632	3,324.64	9,201.06	85.19	135.08	57.19	35.09	88.64
Hospitalization (days)	13	759.57	7	506.38	1,265.96	11.71	63.68	253.19	253.19	316.49
Additional examinations	832	3,133.00	466	2,234.35	5,367.35	49.69	74.58	25.51	17.45	46.25
Medications for asthma control (doses)	32,374	26,769.55	36,204	25,479.19	52,248.74	483.78	385.72	467.73	249.18	634.11
Medications for rescue asthma (doses)	164,962	5,595.74	96,484	4,443.75	10,039.49	92.95	245.65	28.04	5.30	75.57
Medications for asthma (total/doses)	197,336	32,365.29	132,688	29,922.94	62,288.24	576.74	572.48	482.67	250.53	718.78
Medications for rhinitis (doses)^e^	40,442	4,335.02	33,448	5,402.30	9,737.33	90.15	134.58	85.30	33.64	136.15
Medications for respiratory infection (doses)^f^	2,058	4,345.09	1,484	581.68	4,926.78	45.61	124.64	105.26	46.67	159.06
Immunotherapy/Vaccines for allergy(doses+bottles)^g^	852	932.33	468	987.32	1,919.66	17.77	31.40	53.45	46.29	71.28
Environmental control^h^	-	6,014.97	-	6,548.89	12,563.86	116.33	317.35	116.96	46.05	284.65
Transport	-	4,188.24	-	3,291.20	7,479.45	69.25	113.72	44.23	30.38	75.76
Indirect costs										
Absence from work (days)^i^	186	2,359.32	94	2,354.38	4,713.71	43.64	108.54	102.32	38.95	210.44
Absence from school (days)^j^	61	-	48	-	-	-				
Sickness benefit (BAD)^k^	4	14,502.92	2	5,485.38	19,988.30	185.07	948.84	2,631.58	2.602.34	5,760.23

Total cost	-	78,812.23	-	60,639.50	139,451.73	1,291.20	1,298.57	955.78	663.02	1,223.46

Cost related to asthma	-	70,132.11	-	54,655.51	124,787.62	1,155.43	1,305.58	827.46	502.29	1,125.56
Cost related to rhinitis and respiratory infection	-	8,680.12	-	5,983.98	14,64.11	135.77	187.01	102.92	51.05	201.90

^a^ Calculated in relation to the total population studied (n = 108).

^b^ Calculated in relation to the number of individuals that generated the unit of cost in question.

^c^ n = 43 individuals

^d^ n = 33 individuals

^e^ n = 84 individuals

^f^ n = 31 individuals

^g^ n = 32 individuals

^h^ n = 54 individuals

^i^ n = 32 individuals

^j^ n = 18 individuals

^k^ n = 5 individuals/6 sickness benefits (BAD).

**Table 2:** Mean annual cost of asthma in outpatient treatment. stratified by demographic and clinical characteristics.

Category		n (%)	Total		Asthma		Medication for Asthma		p

			Mean	SD	Mean	SD	Mean	SD	
Age (years)	< 20	21 (19.4)	744.60	175.82	601.58	158.36	234.55	109.62	0.000^a^
	20–59	61 (56.5)	1,584.39	966.73	1,461.20	964.47	692.66	366.14	0.000^b^
	≥ 60	26 (24.1)	984.67	287.72	483.58	258.54	601.50	179.45	
Sex	Male	29 (26.8)	1,522.99	1,030.99	832.08	1,032.46	553.95	332.62	0.52^a^
	Female	79 (73.2)	1,186.32	637.22	608.07	629.19	591.80	295.19	0.40^b^
Family income	≤ 1 minimum wage	25 (23.1)	1,315.72	553.62	709.37	560.46	731.36	388.32	0.58^a^
	> 1 to 3 minimum wages	53 (49.1)	1,239.81	754.53	637.39	750.85	524.78	268.44	0.38^a^
	> 3 minimum wages	30 (27.8)	1,318.38	919.68	696.35	911.32	576.24	294.78	
Education level	Elementary school	76 (70.4)	1,164.34	592.57	611.44	591.90	556.90	263.78	0.92^a^
	High School	25 (23.1)	1,614.84	1,131.14	827.46	1,126.16	583.42	358.14	0.55^b^
	Higher education	7 (6.5)	1,289.27	780.29	715.99	790.14	843.79	476.14	
Asthma duration	≤ 10	36 (33.4)	647.43	872.90	345.53	875.32	256.91	321.30	0.30^a^
	> 10–20	26 (24.1)	602.62	614.11	294.57	598.50	268.31	305.28	0.008^b^
	> 20	46 (42.5)	651.47	756.80	348.69	752.14	330.08	290.30	
Asthma severity ^c^	Intermittent	42 (38.9)	432.12	487.14	224.10	472.31	161.61	130.37	
	Mild	24 (22.2)	448.34	267.24	220.73	237.82	217.85	163.01	0.000^a^
	Moderate	31 (28.7)	656.08	888.60	345.91	886.33	283.11	171.84	0.000^b^
	Severe	11 (10.2)	1,403.84	1,180.52	790.73	1,184.74	750.39	570.27	
Asthma control ^c^	Controlled	53 (49.1)	419.48	175.56	212.12	171.66	193.14	118.63	0.000^a^
	Partially controlled	42 (38.9)	637.54	743.01	326.06	738.18	280.76	212.00	0.000^b^
	Uncontrolled	13 (12.0)	1,283.75	1,225.45	713.82	1,234.49	611.06	554.65	
Nutritional status	Normal weight	44 (40.7)	1,027.68	626.53	510.74	609.78	398.82	160.62	0.001^a^
	Overweight	35 (32.4)	1,259.93	575.37	675.81	566.16	626.19	322.12	0.000^b^
	Obese	29 (26.9)	1,674.86	1,066.88	897.98	1,077.41	805.24	399.63	
Rhinitis	Yes	90 (83.3)	636.06	749.83	325.99	746.12	261.23	279.86	0.03^a^
	No	18 (16.7)	649.85	843.33	377.69	838.13	438.75	379.39	0.0001^b^

MW: minimum wage

^a^ Comparisons related to the cost of isolated asthma.

^b^ Comparisons related to the cost of medications for asthma.

^c^ Comparisons related to asthma control and severity in the 2^nd^ and 1^st^ evaluations.

Three patients were hospitalized for asthma (mean of 4.3 days/patient) in the first six months and two (mean of 3.5 days/patient) in the final six months of the study. All the hospitalizations occurred in the hospital of the university, in the SUS.

Eighteen of the 24 students were absent from school and 28 patients were absent from work because of asthma. Including the four individuals who were absent from work to take care of their children, 32 workers (78.0% of the individuals working outside the home) were absent because of asthma. In the first evaluation, four patients were receiving sickness benefit (BAD). In the second evaluation, two had been retired because of asthma, one lost his job, and two were still receiving BAD. The estimated annual cost related to absenteeism was U$24,702.01, the highest proportion because of BAD (Table 1).

The greater severity and worse control of asthma were confirmed as independent factors associated with the higher cost of the disease. Sex had a trend of influence as an independent factor, and weight alone was not an independent factor of cost of asthma in the final result, unlike severity and control (Table 3). The Tornado diagram (Figure) shows the total cost variation based on the 25th-75th percentiles around the median of the different cost units. It shows the significant potential impact of cost variations associated with medications for asthma, environmental control measures, extended leave (BAD), and medications for rhinitis on the total cost of the disease.

**Table 3:** Multivariate analysis for independent factors on the cost of asthma in outpatient treatment.

Variable	Coefficient (95%CI)	p
Age	-0.0003862 (-0.006028–0.0052556)	0.89
Sex	0.2406721 (0.0070797–0.4742645)	0.04
Education level	-0.0975651 (-0.3368083–0.141678)	0.42
Monthly income	0.0012074 (-0.0997113–0.102126)	0.98
Weight	-0.2074344 (-0.423744–0.0088752)	0.06
Asthma severity	0.1637397 (0.0322054–0.295274)	**0.01**
Asthma control	0.6330797 (0.288369–0.9777903)	**0.000**

The values of p in bold indicate the independent factors on the cost of asthma.

**Figure:**
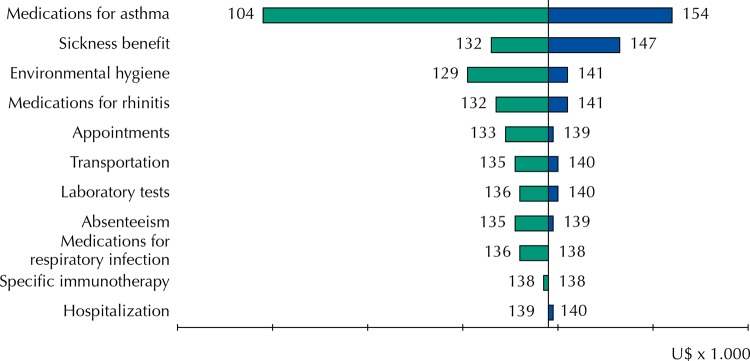
Sensitivity analysis for variations of the different cost units of asthma in outpatient treatment (Tornado diagram).

## DISCUSSION

Few studies are published in Brazil with primary data on the costs of chronic diseases, and most of them refer to cardiovascular diseases, such as chronic coronary artery disease^20^ and congestive heart failure^3^. A recent systematic review has included only three studies on the costs of asthma in Brazil, all focusing only on severe asthma, showing the scarcity of data in the country^25^. Our study provides an estimate of the cost of bronchial asthma in a real-life setting with outpatients with different levels of severity and control, not yet described in Brazil.

From the clinical point of view, the approach used resulted in desirable changes in the severity of the patients, as well as improvement of the control in part of them.

The different methods used in the treatment and valuation of the costs are a problem when comparing results between different countries. Considering the PPP-adjusted values, the estimated total annual cost for asthma per patient in our study was 50.0% higher than the mean cost in the Asia Pacific region^16^. On the other hand, it accounted for just over half of the estimated cost of asthma in the United States^26^ and in the EU^2^ (59.8% and 55.0%, respectively).

Asthma and rhinitis are often associated and influence each other. Studies indicate that 75.0% to 80.0% of asthmatics have rhinitis and 40.0% to 50.0% of individuals with allergic rhinitis or nonallergic rhinitis with eosinophilia syndrome have bronchial hyperresponsiveness^a^. In our study, the coexistence of asthma and rhinitis was high and the exclusive cost of asthma was higher in patients without rhinitis. This is probably due to the greater severity of asthma observed in patients who did not have this association in the study population. Respiratory infections such as sinusitis, otitis media, and pneumonia are also more frequent in these patients^6^ ^,^ ^7^ ^,^ ^a^, which contributes with the associated costs. The incremental cost related to rhinitis or RI was 11.7%, with a total mean cost related to asthma of U$1,155.43/patient-year (SD = 2,232.55), reaching values of U$1,291.22 (SD = 1,298.56) for asthma associated with rhinitis or RI. Direct costs accounted for the highest proportion, and the cost of medications was nearly half the cost of asthma.

At the time of data collection, 30.0% of the patients were given free asthma control medication in the SUS, which directly affected the budget of most families. The total average annual cost had a significant impact on family economy, corresponding to 11.6% of the annual MFI. This value corresponded to 9.0% of the Brazilian gross domestic product *per capita* in 2012^d^. In a study that has evaluated patients with severe asthma in Bahia, the direct cost of the disease corresponded to 24.0% of the MFI in the year before the inclusion of the patients in a structured asthma program. The free supply of medication reduced this proportion to 2.0% of the MFI^13^.

The more severe or worse the asthma control, the greater its cost^1^ ^,^ ^18^ ^,^ ^27^. The costs of severe asthma were 128%, 259%, and 253% higher in relation to moderate, mild, and intermittent asthma, respectively. They were also higher in uncontrolled asthma compared to partially controlled (> 19.0%) and controlled (> 37.0%) asthma. Proportionally to MFI, severe asthmatics cost two to four times more when compared to patients with moderate, mild, or intermittent asthma. Similarly, uncontrolled asthmatics had costs proportional to their MFI of four to six times more than patients who had controlled or partially controlled asthma. Patients with severe and moderate asthma, as well as those with uncontrolled asthma, had reduced MFI during the study. Only among controlled or mild asthmatics did MFI increase, despite the small reduction of the Brazilian *per capita* GDP between 2011 and 2012^d^. Although our study was not designed for this, the findings suggest that the more severe or worse the asthma control, the greater its influence on the work capacity and productivity of families, which seem to increase with better control of the disease.

The cost of uncontrolled asthma was also higher in a study that has compared the direct costs of treatment of patients with controlled and uncontrolled asthma in a tertiary unit in São Paulo^22^. This happened mainly because the patients went more to the emergency room and were hospitalized more frequently, which did not happen in our study. Nevertheless, we had higher costs in this subgroup. Uncontrolled patients also had lower family income, as we have observed in the study population. In a reference center for severe asthma in Bahia, 74.0% of the patients had MFI below the minimum wage^21^. The same group showed a significant reduction in the direct costs of severe asthma after controlling the disease^13^.

Comorbidities are common in adults with asthma and may have an impact on their costs^14^. The increase in overweight/obesity in western societies is associated with an increase in asthma prevalence and severity and also worse asthma control^5^ ^,^ ^11^ ^,^ ^19^. Our results point to higher costs in obese patients than in asthmatics with overweight (> 33.0%) and normal weight (> 76.0%). This is particularly worrying considering that, in addition to the high asthma prevalence in Brazil, the 2012 data of the VIGITEL research showed that 64.3% of Brazilian adults were overweight (48.5%) or obese (15.8%)^e^ ^,^ ^f^. These values increased to 70.4%, 52.5%, and 17.9%, respectively, in 2014^f^. Structured programs to address asthma associated with programs to combat overweight or obesity may be useful in primary care for better asthma control and associated cost reduction in Brazil.

Asthma can have consequences on work capacity, and absenteeism is often underestimated by public health policy makers. Absenteeism is influenced by demographic variables, level of job satisfaction, organizational characteristics of the institution, and content of the activity in question^17^. Our population had a great diversity of occupations and places of work, which prevent us from performing any analysis on these factors. Almost 80% of the workers in this study were absent from work and three-quarters of the students missed classes exclusively from asthma. The estimated total annual cost related to all sick leaves was 17.7% of the estimated total annual cost. The indirect cost was low compared to other studies^4^, probably because of the lower income range of our patients in relation to developed countries.

Our study has limitations. The population studied does not represent the population of Brazilian asthmatics, but rather the population of asthmatics treated at the health unit in question. Participants accounted for one-third of the total number of asthmatics followed and their inclusion happened consecutively to minimize selection biases. Another limitation refers to the lack of reliable data on socioeconomic distribution and the proportions of severity levels of Brazilian asthmatics. Thus, the studied population does not necessarily represent the national reality. The treatment received by these patients does not reflect the routine of public services, since it is a secondary unit of a university, which follows recent international guidelines. Its structural and operational characteristics promoted good adherence to treatment and a small number of hospitalizations, possibly underestimating the cost related to the disease.

The estimated annual cost of asthma in the study population had a considerable impact on the family budget and it was higher in adult patients with more than 20 years of disease, in obese or overweight individuals, and in those with more severe asthma or with worse asthma control. The cost units with the greatest potential impact on total cost were medications for asthma, extended leave, environmental control measures, and medications for rhinitis.

Studies that size the socioeconomic impact of asthma may provide a better basis for the decision-making of health policy makers, particularly important in a scenario of resource scarcity. Our results are an estimate of the cost of treating asthma at a secondary level in the SUS, assuming that the treatment based on GINA represents the ideal approach to the disease. Studies with the same methodology can be replicated in different regions and in units with different levels of health care to expand the knowledge about real costs in Brazil.

## Footnotes

a Global Initiative for Asthma. 2017 GINA Report. Global strategy for asthma management and prevention; 2017 [cited 2017 Dec 1]. Available from: http://ginasthma.org/2017-gina-report-global-strategy-for-asthma-management-and-prevention/

b Ministério da Saúde (BR). Medicamentos gratuitos para asma ajudaram a reduzir em 16% as internações. Brasília (DF); 2014 [cited 2017 Nov 3]. Available from: http://www.brasil.gov.br/saude/2013/06/medicamentos-gratuitos-para-asma-ajudam-a-reduzir-internacoes-no-sus

c The World Bank. PPP conversion factor, private consumption (LCU per international $); 2014 [cited 2017 Dec 1]. Available from: https://data.worldbank.org/indicator/PA.NUS.PP?locations=BR

d The World Bank. GDP *per capita* (current US$; 2014) [cited 2017 Dec 1]. Available from: https://data.worldbank.org/indicator/NY.GDP.PCAP.CD

e Ministério da Saúde (BR), Secretaria de Vigilância em Saúde, Departamento de Vigilância de Doenças e Agravos não Transmissíveis e Promoção da Saúde. Vigitel Brasil 2012: vigilância de fatores de risco e proteção para doenças crônicas por inquérito telefônico. Brasília (DF); 2013 [cited 2017 Nov 3]. Available from: http://pesquisa.bvsalud.org/bvsms/resource/pt/mis-37272

f Ministério da Saúde (BR), Secretaria de Vigilância em Saúde, Departamento de Vigilância de Doenças e Agravos não Transmissíveis e Promoção da Saúde. Vigitel Brasil 2014: vigilância de fatores de risco e proteção para doenças crônicas por inquérito telefônico. Brasília (DF); 2015 [cited 2017 Nov 3]. Available from: http://pesquisa.bvsalud.org/bvsms/resource/pt/mis-37130
